# Single-stranded DNA binding proteins influence APOBEC3A substrate preference

**DOI:** 10.1038/s41598-021-00435-y

**Published:** 2021-10-25

**Authors:** Amber L. Brown, Christopher D. Collins, Secily Thompson, Margo Coxon, Tony M. Mertz, Steven A. Roberts

**Affiliations:** School of Molecular Biosciences and Center for Reproductive Biology, Washington State University, Pullman, WA USA

**Keywords:** Cancer, Cancer genomics, Biochemistry, DNA, Enzymes, Proteins

## Abstract

The cytidine deaminase, APOBEC3A (A3A), is a prominent source of mutations in multiple cancer types. These APOBEC-signature mutations are non-uniformly distributed across cancer genomes, associating with single-stranded (ss) DNA formed during DNA replication and hairpin-forming sequences. The biochemical and cellular factors that influence these specificities are unclear. We measured A3A’s cytidine deaminase activity in vitro on substrates that model potential sources of ssDNA in the cell and found that A3A is more active on hairpins containing 4 nt ssDNA loops compared to hairpins with larger loops, bubble structures, replication fork mimics, ssDNA gaps, or linear DNA. Despite pre-bent ssDNAs being expected to fit better in the A3A active site, we determined A3A favors a 4 nt hairpin substrate only 2- to fivefold over linear ssDNA substrates. Addition of whole cell lysates or purified RPA to cytidine deaminase assays more severely reduced A3A activity on linear ssDNA (45 nt) compared to hairpin substrates. These results indicate that the large enrichment of A3A-driven mutations in hairpin-forming sequences in tumor genomes is likely driven in part by other proteins that preferentially bind longer ssDNA regions, which limit A3A’s access. Furthermore, A3A activity is reduced at ssDNA associated with a stalled T7 RNA polymerase, suggesting that potential protein occlusion by RNA polymerase also limits A3A activity. These results help explain the small transcriptional strand bias for APOBEC mutation signatures in cancer genomes and the general targeting of hairpin-forming sequences in the lagging strand template during DNA replication.

## Introduction

APOBEC3 (“apolipoprotein B mRNA editing enzyme, catalytic polypeptide-like”-3) family enzymes are cytidine deaminases that function as innate immune response factors through their binding and deamination of viruses and transposons, which repress the replication of these elements^1–3^. APOBECs convert cytidine (C) in single-stranded DNA (ssDNA) to deoxyuridine (dU), which either templates for adenosine causing a C to thymidine (T) mutation or is subsequently converted to an abasic site that leads to an C to T or C to guanosine (G) mutation depending on the polymerase used for bypass^4–6^. These enzymes become dysregulated during tumorigenesis, especially in cervical, bladder, breast, lung, and head and neck cancers, and aberrantly mutate the human genome^7–11^. Consequently, APOBEC3 enzymes are the second-most prominent cause of mutation in sequenced tumors^7,12^.

Of the seven APOBEC3 proteins, A3A, APOBEC3B (A3B), and APOBEC3H haplotype I (A3H-I) have been implicated in contributing to cancer mutations^13–17^. A3B is often highly expressed in the nucleus of many cancers and its expression level predicts critical outcomes of estrogen receptor positive breast cancer patients^18^, indicating that this protein plays a critical role in the pathology of the disease, possibly mediated through its induction of mutations. Despite its pan-cellular localization and relatively low abundance compared to other APOBECs^8,10,14,17–19^, we and others have provided evidence that A3A is the predominant APOBEC contributing to cancer mutation^5,13,17,20,21^. An over-representation of mutations at the preferred motifs for A3A activity (TTCA sequences, where A = adenine) is observed in many primary human tumors^13,16^. The number of APOBEC-signature mutations significantly correlates with A3A expression in both primary tumors and breast cancer cell lines^17^. In addition, A3A often provides the majority of cytidine deaminase activity and APOBEC-signature mutations in breast cancer cell lines (even in the presence of A3B and A3H-I) in part due to its stronger cytidine deaminase activity and unique resistance to RNA inhibition^17,22^.

Within tumor genomes, APOBEC-signature mutations are non-uniformly distributed, showing a strong DNA strand bias associated with replication direction and enrichment in hairpin-forming sequences and tRNA genes^13,23–27^. These distributions of A3A-induced mutations indicate that substrate characteristics beyond the already defined sequence preferences^28–32^ modulate A3A activity. Biochemical studies of substrate preferences have been instrumental in understanding the targeting of mutations caused by the related APOBEC, AID, to transcription intermediates formed in immunoglobulin loci. These efforts initially highlighted the structural loops required for AID WRC sequence specificity^33^, determined that AID (and by extension most other APOBECs) is ssDNA specific^34–36^, and identified roles for transcription^37^, R-loops^38^, G-quadruplexes^39^, RPA^40^, and RNA exosome complex activity in allowing AID to deaminate both strands of a transcription unit^41^. Biochemical activities of A3A have been similarly characterized in vitro. These activities include TC sequence specificity^28–31^ even with damage directly upstream of the deaminated C^42^, ssDNA specificity^43^, activity at 5-methyl C^43,44^, and ability to deaminate transcribed DNA^29,31,45^, DNA synthesis intermediates^45^, as well as RNA substrates^46–48^. Moreover, crystal structure analysis of A3A has revealed how the enzyme’s preferred sequence is mediated by specific amino acids which also contribute to the bending of ssDNA into the A3A active site^30,49,50^. As a consequence, A3A prefers hairpin substrates in vitro^13,17,51^, presumably due to greater binding affinity to the already bent ssDNA^30^. This known specificity likely underlies recurrent mutations at hairpin-forming sequences in cancer genomes that have been predicted to be deaminated by APOBECs 38- to 265-fold over non-hairpin-forming sequences^13,25^. However, A3A has been shown to deaminate ssDNA within replication intermediates in cells^52,53^ and related APOBECs can deaminate transcription intermediates^37,54–58^, raising the question as to why replicative asymmetries dominate transcriptional asymmetries among APOBEC-signature mutations in cancer genomes.

To determine how DNA substrate characteristics influence the distribution of A3A-induced mutations observed in vivo, we measured activity of A3A on several ssDNA substrate types. We recapitulated the previously reported preference for hairpins over linear ssDNA substrates^13,17,30^. However, highly purified A3A only favored hairpin-forming sequences 2- to fivefold (depending on A3A concentration) over linear ssDNA. A3A activity on linear ssDNA was more severely reduced than on hairpin substrates when incubated in the presence of whole-cell lysate or RPA, indicating cellular ssDNA binding proteins likely out-compete A3A for longer ssDNA substrates. This result highlights that substrate specificities for other ssDNA binding proteins likely enhance the preference of A3A-induced mutations for hairpin-forming sequences in tumors. Additionally, we determined that A3A deamination activity is low on ssDNA bubble substrates, like those found during transcription, using an in vitro transcription assay. These results indicate that DNA structure and competition with other ssDNA binding proteins are important factors in predicting at-risk locations in the human genome for A3A-induced mutagenesis.

## Results

**Figure 1:**
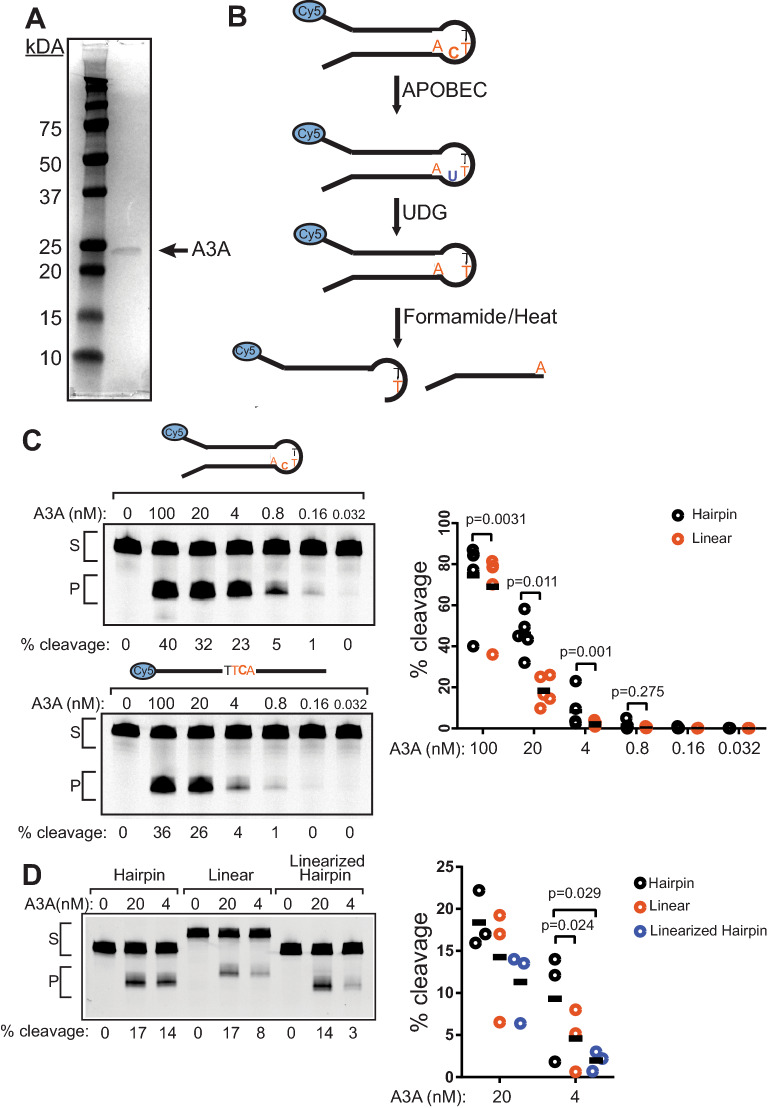
APOBEC3A prefers to deaminate hairpins over linear ssDNA. (**A**) Coomassie stained SDS-PAGE gel showing the purity of C-terminally Strep-tagged A3A (23 kDa) overexpressed in and purified from HEK293T cells. (**B**) Schematic of the in vitro deaminase assay. (**C**) Representative gel images of the activity of decreasing concentrations of purified A3A incubated with 250 nM hairpin and linear substrates in vitro for 30 min at 37 °C in the presence of uracil DNA glycosylase. S denotes substrate band; P denotes the product band. Quantification of 5 replicate experiments comparing A3A activity on hairpin and linear substrates. Horizontal bars indicate mean values. P-values were determined by Ratio paired t-tests. (**D**) 20 nM A3A, 4 nM A3A or no A3A was incubated with 250 nM hairpin or linear substrates. Reactions were performed and denoted as in 1C. Shown is a representative gel image and the quantification of 3 independent experimental replicates (horizontal bars represent mean values for each condition). P-values were determined as in (**C**). Full-length gel images for 1C and 1D are presented in Fig. S4.

To investigate the enzymatic requirements of A3A’s substrate preferences, we measured the activity of purified A3A (Fig. 1A) on multiple ssDNA-containing substrates by in vitro deaminase activity assays. Cytidine deaminase activity at the TTCA sequence in each substrate creates a deoxyuridine base, which is converted to a heat-labile abasic site by the activity of uracil-DNA glycosylase (UDG), resulting in shorter ssDNA fragments (Fig. 1B) that are resolved from uncleaved substrate on a denaturing polyacrylamide gel. We initially compared A3A activity between linear ssDNA substrate and a hairpin-forming oligonucleotide. The sequence of our hairpin substrate consists of a hotspot for APOBEC mutagenesis in breast cancers^25^ likely due to A3A’s affinity for U-shaped ssDNA present in hairpin structures^30,49,50^. We first incubated A3A at a range of concentrations with a constant concentration of substrate to see how deaminase activity would titrate on these substrates (Fig. 1C). We found that activity on the linear substrate decreases faster than it does for hairpins. At most, we see ~ fivefold preference for hairpin over linear substrate with 4 nM A3A.

Previous comparative measurements of A3A activity on hairpin and linear ssDNA substrates have reported strong A3A preferences for hairpin structures^13^, suggesting that differences in the sequence composition of the hairpin may be important effectors of A3A activity. To test this, we incubated our hairpin substrate alongside a previously evaluated mutation hotspot hairpin sequence (NUP93 substrate) and a substrate with the 3′ arm of the stem loop replaced with poly-A to prevent hairpin formation (NUP93-noHP)^13^ (Fig. S1). A3A displayed a similar ~ 2–5-fold preference for each hairpin-forming sequence compared to their corresponding linear ssDNA substrate (Fig. 1D and Fig. S1). This indicates that a broader sequence content of the hairpin is unlikely to be a significant factor in determining A3A activity, but instead the general conformation of the stem-loop structure of the hairpin is the major activity determinant.

### APOBEC3A prefers small hairpin loops with target C in central positions

**Figure 2:**
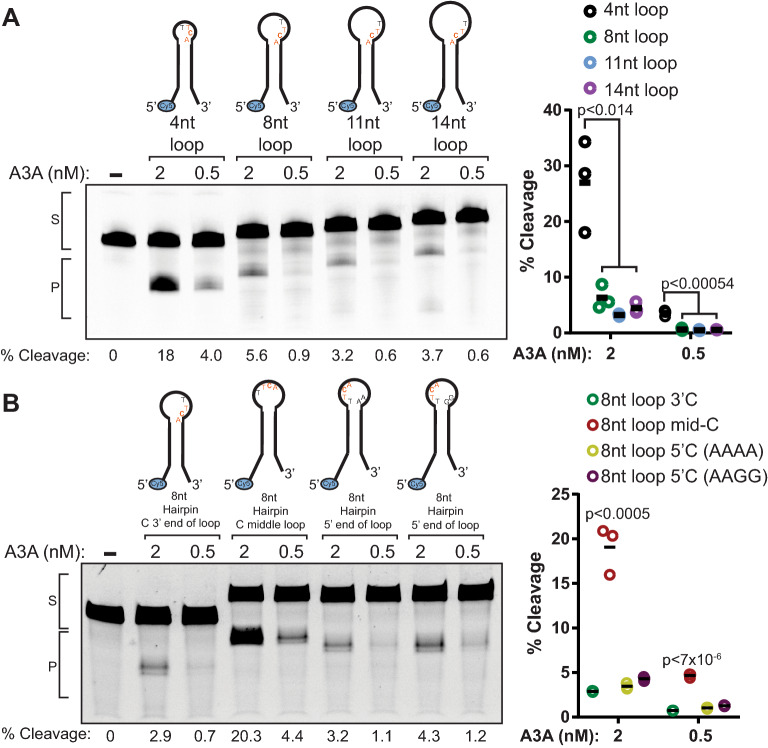
APOBEC3A prefers stem loops with 4nt loops, and target C in the middle of the loop. (**A**) Deaminase assay of 2 nM A3A, 0.5 nM A3A or no A3A incubated with 100 nM hairpins with 4, 8, 11, or 14 nt loops for 1 h at 37 °C before processing as in Fig. 1C. (**B**) Deaminase assay as in (**A**) using 8 nt hairpins with the target C in the 3′-most (8nt loop 3′C), middle (8nt loop mid-C), or 5′-most (8nt loop 5′C) position in the loop for 3 h at 37 °C before processing as in Fig. 1C. Two versions of the 8nt loop 5′C hairpin are included, where the sequence of the 3′ portion of the loop is either AAAA [8nt loop 5′C (AAAA)] or AAGG [8nt loop 5′C (AAGG)]. S denotes substrate band; P denotes the product band. Full-length gel images for 2A and 2B are presented in Fig. S5. Quantifications of the percent cleavage for 3 replicate experiments of (**A**) and (**B**) are shown. Horizontal bars indicate mean values. P-values were determined by t-test.

To further characterize the effect hairpin structure has on A3A activity, we conducted deaminase activity assays with hairpin substrates with varying loop sizes. The hairpin substrates have the target TTCA sequence at the same 3′ position in the loop, with the terminal A being the first base within duplex DNA. The loop size in each substrate was increased by the addition of poly-adenine, for which A3A has low binding affinity^30^, upstream of the target site towards the 5’ end of the loop. When incubated with 20 nM A3A, hairpin substrate deamination was decreased fourfold, sixfold, and fivefold on the 8 nt loop, 11 nt loop, and 14 nt loop, respectively (Fig. 2A), indicating that the larger loops either reduce A3A’s binding affinity or catalytic efficiency.

To determine if the position of the target C within hairpin loops affects A3A activity, we conducted deaminase activity assays with 2 and 0.5 nM A3A, and 8 nt loops with the target C shifted to the 3′-most end, the middle, and the 5′-most end of the loop. Again, shifting of the target C was accomplished by insertion of A nucleotides around the target TTCA (Fig. 2B). We observed that moving the target C to the middle of the 8 nt loop increased A3A activity, while moving the target C to the 5′-most position reduced A3A cytidine deamination. However, for the 5′-most target C, the TT in TTCA could potentially pair to AA on the opposite side of the loop, making it double stranded and reducing the loop size from 8 to 4 nt. Therefore, we tested a variant of this substrate where the AA dinucleotides at the 3′ most position of the loop were replaced with GG to prevent unwanted annealing within the loop. A3A activity toward this substrate was similarly reduced compared to the loop with the centrally positioned C, consistent with previous results indicating that A3A favors hairpin loops with cytidines located in the center^13^.

### ssDNA bubbles are disfavored APOBEC3A substrates

**Figure 3:**
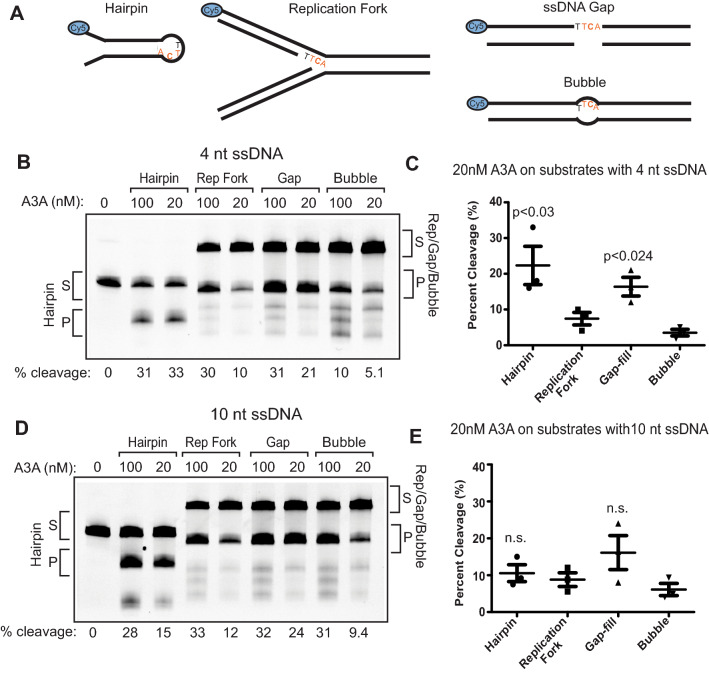
APOBEC3A prefers hairpin substrates over other structures that are possible sources of ssDNA. (**A**) Graphical representation of ssDNA substrates tested. All substrates contained a TTCA motif and were 5′ Cy5 tagged. Structures with double-stranded regions were confirmed prior to use (Fig. S2). (**B**) Deaminase assay of 100 nM A3A, 20 nM A3A, or no A3A was incubated with 20 nM hairpin, replication fork, ssDNA gap, or bubble substrates containing 4 nt spans of ssDNA. Assays were performed and processed as in Fig. 1C. S denotes substrate band; P denotes the product band. (**C**) Quantification of 3 deaminase assay replicates was conducted as described in (**B**). P-values indicate significant differences in A3A activity comparing the hairpin and ssDNA gap substrates pairwise to the replication fork and bubble substrates by t-test. Deamination efficiency was displayed as percent cleavage of each substrate by 20 nM A3A. Error bars indicate standard error of the mean. (**D**) Deaminase assay of 100 nM A3A, 20 nM A3A, or no A3A with 20 nM of hairpin, replication fork, ssDNA gap, or bubble substrates with longer spans of ssDNA (10 nt). Assays were performed and processed as in Fig. 1C. S denotes substrate band; P denotes the product band. (**E**) Quantification of 3 replicate deaminase assays was conducted as in (**D**) and displayed as in (**C**). n.s. indicates no statistical difference was observed in activities among substrates. Full-length gel images for 3B and 3D are presented in Fig. S6.

In addition to hairpin-forming DNA sequences, several transient DNA structures represent abundant sources of genomic ssDNA that could be targeted by A3A. We therefore tested A3A’s ability to deaminate substrates that mimic a replication fork with lagging strand ssDNA, a ssDNA bubble that mimics a transcription bubble, and a small ssDNA gap substrate (Fig. 3A). Each substrate was 5′ Cy5 tagged and contained a single TTCA sequence within a centrally located 4-nucleotide ssDNA region. The replication fork, ssDNA bubble, and ssDNA gap substrates were annealed, gel purified, and respective structures confirmed via restriction digest prior to use (Fig. S2). The substrates (20 nM) were incubated with 100 nM or 20 nM purified A3A for 30 min at 37 °C and the deamination product was resolved from the un-cleaved substrate via denaturing gel electrophoresis. A3A deaminated the hairpin substrate 1.5-, 3-, and sixfold more efficiently than the ssDNA gap, replication fork, and the bubble substrate, respectively (Fig. 3B,C).

Previous reports have indicated that A3A activity can be limited on ssDNAs smaller than 5 nt^29,30,59^. Therefore, the 4 nt ssDNA present in our replication fork, ssDNA gap, and bubble substrates may be size exclusionary. We therefore recreated each substrate with an additional 6 nt of ssDNA 5′ of the target TTCA sequences and repeated the deaminase assays (Fig. 3D,E). We found that A3A activity did not significantly change on the larger ssDNA gaps or replication fork substrates, indicating that additional ssDNA 5′ of the target C does not influence A3A activity. This is consistent with crystallographic data showing limited contact between the A3A active site and nucleotides in the − 3 position^49,50^. Interestingly, increasing the amount of ssDNA 5′ of the deamination site increased A3A activity on bubble substrates. This result is consistent with previous reports^29,59^, which interpreted the increase in activity as A3A requiring longer ssDNA regions. However, the higher level of deamination on the 4 nt ssDNA gap substrate compared to the 4nt bubble instead suggests that the presence of an intact non-deaminated strand in the substrate imposes structural constraints that lessen the ability of A3A to catalyze the deamination reaction. This structural constraint appears to be weaker in larger bubbles, which behave more similarly to longer ssDNA gaps. We quantified the differences in A3A activities on these substrates by calculating specific activities (Table 1).

**Table 1:** Specific activities of A3A on various ssDNA substrates.

Substrate	ssDNA length (bp)	Specific activity (nmol product/min/nmol A3A)	Standard deviation	Measurements
Hairpin	4	0.0133	0.0037	6
Hairpin	10	0.0046	0.0016	3
Linear	41	0.0082	0.0030	4
Replication fork	4	0.0031	0.0008	3
Replication fork	10	0.0035	0.0010	3
ssDNA gap	4	0.0034	0.0030	7
ssDNA gap	10	0.0061	0.0024	3
Bubble	4	0.0008	0.0006	6
Bubble	10	0.0023	0.0009	3

### APOBEC3A activity is reduced on linear substrates when incubated with cell lysate

**Figure 4:**
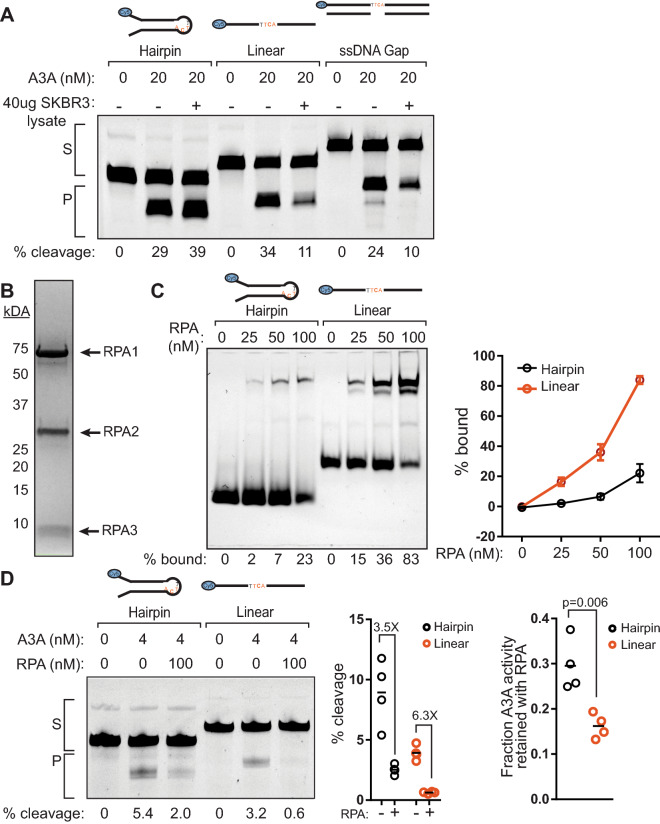
APOBEC3A activity is reduced more severely on linear substrates than on hairpins in the presence of whole cell lysate or RPA. (**A**) Deaminase assay of 20 nM A3A or no A3A incubated with 20 nM hairpin, linear, or gap-fill substrates with or without whole cell lysate. Substrates were preincubated with 40 µg of SKBR3 whole cell lysates or buffer for 1 h at 37 °C, then A3A was added to 20 nM final concentration, and incubated for an additional 30 min at 37 °C prior to processing as in Fig. 1C. S denotes the substrate band; P denotes the product band. The results are representative of three independent experiments. (**B**) Coomassie stained SDS-PAGE gel of purified human RPA. (**C**) Electrophoretic mobility shift assay of 0, 25, 50, and 100 nM RPA incubated with 50 nM hairpin or linear ssDNA substrate. Three replicate experiments were quantified to determine the percent of each substrate bound. Dots and error bars indicate mean values and standard deviation. (**D**) 4 nM A3A was incubated with 50 nM hairpin or linear ssDNA substrate at 37 °C for 5 or 15 min, respectively, in the presence or absence of 100 nM RPA. The percent substrate cleavage in the presence and absence of 100 nM RPA as well as the fraction of A3A deaminase activity remaining after RPA addition for the hairpin (black dots) and linear substrates (red dots) was quantified from 4 experimental replicates. Horizonal bars indicate mean values. P-values were determined by t-test. Full-length gel images for 4A and 4D are presented in Fig. S7.

A3A has been shown previously to greatly prefer hairpin substrates over linear ssDNA substrates^13,17,30,51^, which results in recurrent mutation of hairpin-forming sequences in human tumors^13,25^. However, the fold-preference for hairpin substrates has varied substantially under different experimental conditions, with assays conducted utilizing cell lysates showing the greatest hairpin preference^13^. We have found that purified A3A prefer hairpins over linear substrates, but that the preference is only 2- to fivefold depending on A3A concentration (Fig. 1D). We hypothesized that the presence of proteins in cell lysate may enhance A3A preference for hairpin over linear substrate; these additional proteins may compete with A3A for binding of linear ssDNA, but not hairpin DNA. To test this, we performed a deaminase assay with or without the addition of 40 µg whole cell lysate from the breast cancer cell line SKBR3. We pre-incubated the hairpin, linear, or ssDNA gap substrates with the cell lysate for 30 min at 37 °C to allow any ssDNA-binding proteins to bind the substrates prior to the addition of A3A (20 nM final concentration). Following denaturing gel electrophoresis, we found that deamination of the hairpin substrate was unaffected upon the addition of whole cell lysate, while A3A activities on the linear and the ssDNA gap substrates were reduced 3- and 2.5-fold, respectively (Fig. 4A). Thus, other proteins present in the whole cell lysate appear to compete with A3A for binding of linear and ssDNA gap substrates. However, the bent conformation of ssDNA within the hairpin substrate is resistant to this inhibitory effect.

The identity of ssDNA-binding proteins that preferentially inhibit A3A on linear ssDNA substrates is unknown. Replication Protein A (RPA) is a likely candidate, as it is known to inhibit A3A activity on linear ssDNA substrates^45,60^ and requires approximately 30 nt of ssDNA for high-affinity binding^61,62^. We therefore purified human RPA (Fig. 4B), a heterotrimer composed of RPA1 (70 kDa), RPA2 (32 kDa) and RPA3 (14 kDa) and tested its ability to inhibit A3A cytidine deaminase activity on both hairpin and linear ssDNA. We found that RPA nearly saturated the linear ssDNA substrate, but not the hairpin substrate when in approximately a twofold excess over substrate (Fig. 4C), indicating that RPA binds hairpin substrates less efficiently. The stronger RPA binding of linear ssDNA translated into RPA dramatically decreasing A3A activity 6.3-fold on linear ssDNA. RPA also reduced A3A activity on hairpin substrates, but only by 3.5-fold (Fig. 4D), indicating that RPA binds less strongly to small ssDNA loops in hairpin structures than to linear ssDNA, which allows A3A to maintain activity at sites of DNA secondary structures.

### A3A activity is low on transcription bubbles

**Figure 5:**
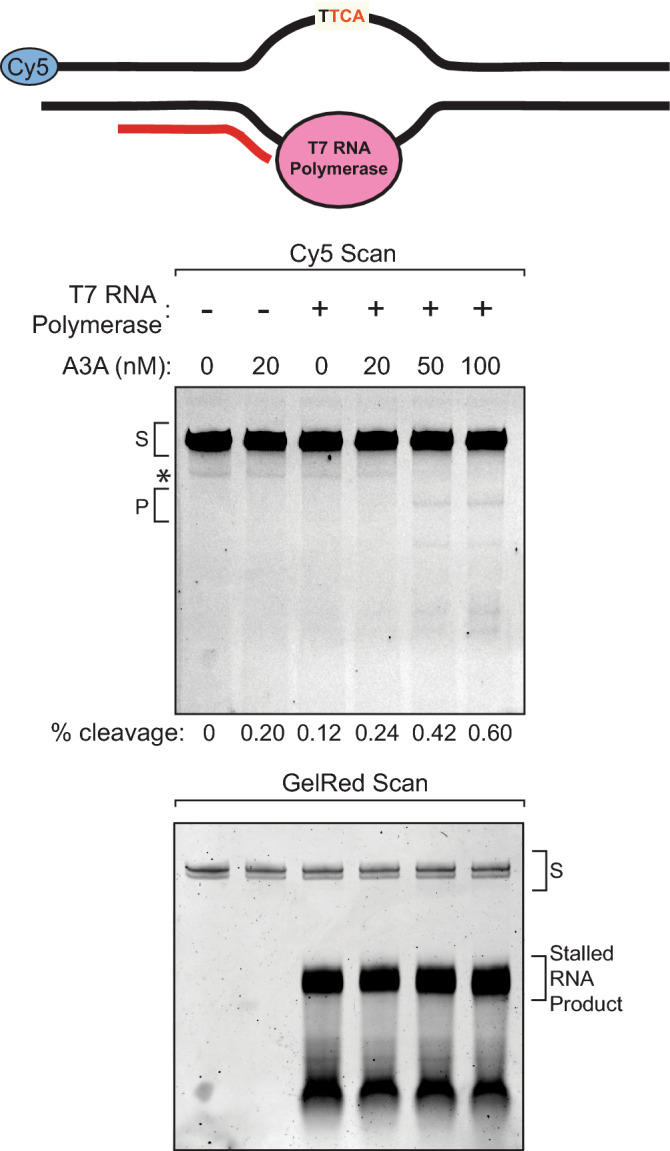
APOBEC3A activity is low on transcription bubbles. Deaminase assay of 0, 20, 50, or 100 nM A3A during in vitro T7 RNA polymerase transcription, stalled at T in AAGT within the transcribed strand (i.e. across from a TTCA on the non-transcribed strand). Reactions were carried out with 50 units of T7 RNA polymerase or an equal volume of 50% glycerol, and GTP, CTP, and UTP but no ATP, to cause the stalling. Reactions were incubated for 24 h at 37 °C in the presence of Uracil DNA Glycosylase, stopped by the addition of Proteinase K and SDS buffer, then heated for 10 min at 95 °C in formamide buffer before separating out product from substrate via denaturing polyacrylamide gels. Gels were imaged for Cy5 fluorescent tags (upper panel) and after staining with GelRed to observed total nucleic acids produced in the reaction (lower panel). S denotes the substrate band; P denotes the product band. Full-length gel images are presented in Fig. S8. Shown is a representative image from 10 independent experiments.

Due to the potential for protein obstruction to hinder A3A’s activity on ssDNA, we evaluated whether other proteins may also help to restrict A3A activity to the lagging strand template during replication. Transcription, like replication, produces ssDNA on the non-transcribed strand and is required for genomic cytidine deamination by AID^37,58^. However, little evidence for transcriptional asymmetry of APOBEC-induced mutations in cancer genomes exists^23,24,26^, indicating that ssDNA within transcription bubbles may be protected from APOBEC cytidine deaminase activity. Our previous data indicates that the small ssDNA bubbles themselves reduce A3A activity. Moreover, transcription bubbles are frequently 14–22 nt in length^63^, indicating that most ssDNA present will be near the synthesizing RNA polymerase, which may further reduce A3A activity. To better understand whether RNA polymerase itself blocks A3A activity, we generated an in vitro transcription system similar to previously described systems utilizing T7 RNA polymerase^29,31,35,37,38,45^, a 90 bp dsDNA substrate, and our purified A3A. To enhance the stability of transcription-associated ssDNA, we designed the substrate to only contain T bases in the transcribed DNA strand downstream of the TTCA deamination target on the non-transcribed strand and excluded rATP from the transcription reaction. The lack of rATP stalls T7 RNA polymerase across from the TTCA deamination target sequence, causing it to remain single stranded as long as T7 RNA polymerase is bound. 0, 20, 50, or 100 nM of purified A3A were incubated with 20 nM dsDNA substrate and 50 units of T7 RNA polymerase at 37 °C for 24 h. A3A was incapable of deaminating the dsDNA alone (Fig. 5, upper panel), or in the presence of stalled T7 RNA polymerase transcription, which was confirmed by the presence of a 21 nt RNA product in a GelRed scan for total nucleotide content (Fig. 5, lower panel). A low-abundance ssDNA oligonucleotide (indicated with * in Fig. 5 upper panel) was observed as a contaminant in our dsDNA T7 RNA polymerase substrate. A3A deaminated this ssDNA contaminant, but not the T7 transcription substrate, indicating that the presence of the T7 RNA polymerase efficiently blocks A3A from directly deaminating ssDNA within a transcription bubble.

Previous findings indicate that A3A can deaminate cytidines during in vitro transcription^29,31,45^. Our in vitro transcription system stalls the T7 RNA polymerase at the first cytidine:guanosine base pair in the substrate such that the A3A target is only ssDNA when it is associated with the T7 RNA polymerase. In contrast, all previous in vitro systems used to investigate A3A-mediated deamination of transcription, T7 RNA polymerase can transcribe past potential A3A-deamination sites. Establishment of extended RNA-loops during this process would allow cytidines within the non-transcribed DNA strand to remain single stranded in the absence of the T7 polymerase. Thus, our data indicate that that A3A is unlikely to actively deaminate cytidines within RNA polymerase-associated transcription bubbles, but instead likely requires the formation of extended R-loops behind the extending polymerase to enable A3A to preferentially deaminate the non-transcribed DNA strand.

## Discussion

Despite having lesser enrichment than other types of cancer mutations in some chromosomal features like late replicating regions and heterochromatin^24,64^, APOBEC-induced mutations are still non-randomly distributed with respect to the lagging strand template^23,24,26,53,65^, tRNA and rDNA genes^27,54,55^, and hairpin-forming sequences^13,25^. However, the underlying mechanisms that contribute to the distribution of APOBEC-induced mutations in many cases are unknown. A3A has been implicated as a major source for APOBEC-signature mutations in human cancers, in part due to its strong preference for deaminating cytidines in hairpin structures, which are also frequently mutated at APOBEC target sequences in tumors. Consistent with previous results, we find that A3A prefers ssDNA within hairpin secondary structures for deamination. Hairpins containing a 4 nt loop were favored over those with larger ssDNA loops as was a centrally positioned target cytidine within 8 nt loops. Among different substrates mimicking potentially physiological sources of ssDNA, A3A prefers substrates with small ssDNA regions in the order of: hairpin > ssDNA gap > replication fork > bubble. Within a cell, ssDNA regions, especially those associated with lagging strand synthesis are likely to be significantly longer. We expect that A3A will exhibit similar activity towards these regions as towards the fully linear ssDNA substrate, unless these regions contain sequences that allow for local secondary structures to form. One potential caveat of these data is the presence of a c-terminal strep-tag on A3A, which could influence A3A activity, however, based on the small size of the tag (8 amino acids), we believe this impact would be small. Supporting this assertion, un-tagged A3A and the c-terminal strep-tagged A3A display similar levels of cytidine deaminase activity on a hairpin substrate with a 4 bp loop (Fig. S9).

Surprisingly, A3A only preferred hairpin ssDNA 2- to fivefold over fully linear ssDNA, a less drastic fold difference than has been previously reported^13^. Favoring of the hairpin structure becomes more pronounced at lower A3A concentrations. Still, the relatively small preference for hairpin substrates over ssDNA substrates suggests that factors beyond A3A’s innate structural preference for hairpin DNA binding likely influence A3A specificity in cells and give rise to the over 200-fold preference for APOBEC-induced mutagenesis in hairpin-forming sequences compared to linear DNA. In particular, hairpins may be less likely to be occluded by ssDNA-binding proteins within a cell, which would enhance A3A specificity for hairpin-forming sites. We found that A3A activity towards cytidines within linear ssDNA was reduced when whole cell extracts from the SKBR3 breast cancer cell line was included in deamination reactions, while activity on the hairpin substrate was unaffected. We initially hypothesized that the increased length of ssDNA in the linear substrates (i.e. 45 nt long) would allow binding of ssDNA-binding proteins whose footprint would be unable to bind to the shorter 4 nt ssDNA contained within the hairpin substrate. However, A3A activity towards a 4 nt ssDNA gap substrate was also reduced by SKBR3 whole cell extracts, indicating that the bent conformation of the 4 nt loop of the hairpin may be the primary barrier to the binding of putative competitor proteins.

The identity of specific protein factors that influence the distribution of A3A-induced mutation are unknown. RPA is one likely candidate as it inhibits the activity of multiple APOBECs, including A3A, on linear ssDNA^45,53,60,66,67^, and binds to a relatively large stretch of ssDNA (~ 30 nt)^61,62^, suggesting that it may be unable to inhibit A3A activity on smaller, structured ssDNA regions. Supporting this, RPA efficiently inhibits A3A activity at near equimolar ratios of RPA to a 40 nt linear ssDNA substrate, while impacting A3A activity on a hairpin substrate with a 4 nt loop to a lesser extent. Thus, the binding of longer ssDNAs, formed during replication or in extended DNA R-loops, may provide protection from mutation, enhancing the preferential mutagenesis on hairpin-forming sequences. RPA typically binds and prevents the formation of DNA secondary structures, protecting the ssDNA from DNA damaging agents like A3A. In cancers, replication stress-induced ssDNA formation can exhaust RPA levels^68^, leaving sections of ssDNA in replication forks unprotected. These long regions of ssDNA can then form hairpins that would otherwise be repressed by RPA binding and further make themselves targetable by A3A. As a result, A3A-induced mutations are enriched at hairpin-forming regions of cancer genomes and show strand bias for replication intermediates.

It is also possible that larger ssDNA binding proteins, like RNA Polymerase occlude A3A binding. In our in vitro transcription assay, a 99 kDa T7 RNA Polymerase is stalled across from the TTCA target site and its proximity likely inhibits the smaller 23 kDa A3A by blocking access to the target site. Transcription of protein coding genes in human cells is carried out by the RNA Polymerase II complex, which is comprised of 10 subunits that together constitute a mass of ~ 500 kDa^69^. The large size of this complex likely occludes A3A and prevents A3A from deaminating and eventually mutating transcription intermediates. Hypothetically, the non-transcribed strand would be single-stranded for a period during transcription and could be deaminated by APOBEC3s. Indeed, there is experimental evidence for A3A deaminating transcription intermediates in vitro^29,31,45^. However, only a small enrichment has been found for APOBEC-signature mutations on the non-transcribed strand in human cancers^23,24,26^. Consistent with this result, we saw nearly undetectable A3A activity on the non-transcribed strand during stalled T7 RNA polymerase transcription. Additionally, A3A activity was also reduced towards a ssDNA bubble substrate that mimicked the static structure of a transcription intermediate. These data suggest that A3A is unlikely to be active at transcription bubbles, especially those directly blocked by the presence of the elongating RNA polymerase. Instead, transcription would likely require the creation of an elongated ssDNA bubble by the formation of an R-loop, allowing A3A sufficient access to the ssDNA for deamination. Consequently, transcriptional strand asymmetry of A3A-induced mutations in human cancers may only be present in DNA regions prone to R-loop formation.

## Methods

### Purification of APOBEC3A

A3A was purified as in^17^. Briefly, HEK293T cells were transduced with lentiviral vectors encoding the *APOBEC3A* gene under control of the Tet repressor. Cell populations were expanded to approximately sixty 10 cm dishes, after which A3A protein expression was induced with doxycycline for approximately 84 h. A3A-expressing cells were then harvested via trypsinization and centrifugation. Lysates were prepared in buffer containing protease inhibitors and DTT, sonicated, and treated with Benzonase. Insoluble materials were removed via centrifugation and filtering. The Strep-tagged A3A was batch bound to Strep-tactin resin and put over a glass econo-column. The column was washed to remove any non-specific binding proteins and then the Strep-tagged A3A was eluted using buffer containing 20 nM d-desthiobiotin. Eluted A3A was further purified through an 1 mL Enrich-Q (BioRad) column. A3A-containing fractions were pooled, concentrated, dialyzed, frozen in aliquots in liquid nitrogen and stored at − 80 °C. Aliquots were not used past 5 freeze/thaw cycles.

### RPA purification

Three subunit human RPA was expressed from plasmid p11d-tRPA(123), (Addgene; 102613), in T7 express *E. coli* (NEB; C2566I). Human RPA was purified following protocols previously described in^70^, using chromatography on subsequent 5 mL HiTrap Blue HP (Cytiva lifesciences), 2 mL hydroxyapatite CHT-type1 (BioRad), and 1 mL Enrich-Q (BioRad) columns.

### Characterization of whole cell extracts from HEK293T expressing A3A

Creation of HEK293T-TETR cells and pTM-664, a lentiviral vector for inducible expression of Strep-tagged A3A, were described previously^17^. The coding sequence of A3A with its natural stop codon (no tag) and A3A intron 3 was cloned into the HincII and EcoRV sites of pENTR1A no ccDB (Addgene, #17398). An LR-Clonase reaction between the resulting plasmid and pTM-637^17^ was used to create pTM-925, a lentiviral vector for inducible expression of untagged A3A. HEK293T-TETR cells were transfected with plasmids *psPAX2* (Addgene, #12260), pMD2.G (Addgene, #12259), and either pTM-664 or pTM-925 to produce lentiviral particles, which were subsequently used to transduce HEK-293T-TETR cells using LentiBlast (OZ Biosciences). Following selection of stable cell populations with 1 μg/ml puromycin, expression of A3A was induced with doxycycline at 50 μg/ml for 72 h prior to harvesting for whole cell lysates. Whole cell lysates were produced by resuspending cell pellets in 500 µL of M-PER lysis buffer and 2 × protease inhibitors then incubating at 4 °C for 30 min with agitation. Lysates were sonicated four times for 15 s. Debris was removed from lysates by centrifugation at 18,000 rcf for 20 min at 4 °C then glycerol was added to 10% (v/v) supernatant. 20 µg whole cell protein lysate was electrophoresed on a Bio-Rad Mini-Protean TGX SDS-PAGE Precast gel before transferring onto a 0.2 µm PVDF membrane via semi-dry transfer on a Trans-Blot Turbo transfer system. Membranes were blocked with 2% ECL-blocking agent (GE Biosciences) in TBST at room temperature for 1 h before probing with either mouse anti-A3A^21^ (gift from Dr. John Maciejowski) or rabbit anti-alpha tubulin (Ab4074) diluted in 1% ECL-blocking agent in TBST overnight at 4 °C. Membranes were probed with a secondary goat anti-mouse IgG HRP (Ab205719) or goat anti-rabbit IgG HRP (Ab97051) antibody. Blots were developed with ECL prime and imaged on a ChemiDoc MP.

### Assembling substrate structures in vitro

The oligonucleotides used were obtained from IDT technologies and sequences are listed in Table S1. To make the 4 nt set of substrates, the following oligos were incubated: Replication fork (ALH256, ALH232, ALH234, ALH235), ssDNA gap (ALH256, ALH234, ALH240), Bubble (ALH256, ALH241). To make the 10 nt set of substrates, the following oligos were incubated: Replication fork (ALH257, ALH232, ALH234, ALH235), ssDNA gap (ALH257, ALH234, ALH240), and Bubble (ALH257, ALH244).

Substrates with complementary oligonucleotides were annealed by mixing the corresponding oligos in buffer containing 50 mM Tris, pH 7.5 and 100 mM NaCl before heating at 95 °C for 5 min and then slowly cooling (1 °C per minute) in a BioRad Thermocycler. Annealing reactions were then concentrated by EtOH precipitation and run out on a large native 5% polyacrylamide gel (1 × TBE), bands imaged on the Biorad ChemiDoc for Cy5, and excised. DNA was isolated from excised bands using the freeze/squeeze method. Briefly, bands were minced and resuspended in 200 µL of 10 mM Tris, 0.1 mM EDTA buffer, and put through 3 cycles of vortexing (20 s), flash-froze in liquid nitrogen, thawed (65 °C) before incubating at 37 °C, and rotated overnight. Gel debris was separated from DNA-containing supernatant using a 1 mL filter column and spinning at 13 k rpm for 10 min at 4 °C, then DNA was EtOH precipitated from the supernatant. The band with the correct annealing product was determined by restriction digest, as double-stranded sections would have a combination of PacI-HF, KpnI or DraI sites, and would only cut if annealed properly (Fig. S2).

### Measurement of deaminase activity

All deaminase activity reactions in this manuscript were conducted with a single preparation of purified A3A. Deaminase activity assays with A3A (with concentrations as indicated), 5 units of Uracil DNA glycosylase (NEB), 20 nM oligonucleotide substrate, 20 mM Tris HCl pH 7.5, 1 mM DTT, and 1 mM EDTA, in a 20 μL volume were incubated for 30 min (unless otherwise indicated) at 37 °C and terminated by the addition of 7 µL stop buffer (10 mM Tris, 1 mM EDTA, 0.5% SDS). Proteinase K (0.05 mg/mL final) was then added and incubated at 37 °C for another 30 min to limit protein binding to substrates during electrophoresis. Alternatively, cytidine deaminase reactions to test the impact of the strep-tag on A3A activity contained 20 µg cell extract from un-transduced HEK293T cells or HEK293T cells expressing un-tagged A3A or C-terminally Strep-tagged A3A and 1 µM oTM-814 and were incubated for 5 min at 37 °C prior to termination as conducted for assays described above with purified A3A. We then added 23.2 µL formamide buffer (39.4% formamide, 7.5 mM EDTA, and 0.010% SDS), and 4.8 µL of 1 M NaOH (0.1 M final) and incubated at 95 °C for 10 min to break abasic sites present in the substrates. Reactions used to evaluate the impact of RPA on A3A activity also contained 10 nM of unlabeled ssDNA that lacks a cytidine. Deaminase activity products were separated from undigested substrate on pre-warmed 15% polyacrylamide gels with 7.9 M urea (7 cm) in 1xTBE buffer at 15 W for 15 min. Gels were imaged on a BioRad Chemidoc using the Cy5 setting. Percent substrate cleaved was determined by quantification of the intensity of the substrate band and cleavage product bands using the Volume tool on BioRad’s Image Lab software version 5.2, and by using the following equation = (Product intensity)/(substrate + product intensity) × 100.

For deaminase assays with the addition of whole cell lysate, 40 µg of SKBR3 whole cell lysate (generated as in^17^) or buffer were pre-incubated at 37 °C for 30 min with the 20 nM substrate in 20 mM Tris HCl pH 7.5, 1 mM DTT, and 1 mM EDTA with 5 units of UDG, in a 19 µL volume prior to the addition of A3A. After pre-incubation, 1 µL of A3A was added to 20 nM final concentration followed by incubation for an additional 30 min at 37 °C and was processed like the other deaminase assays. Similarly, RPA-containing deaminase assays, involved an initial 30 min pre-incubation of the deamination substrate with a twofold excess of RPA relative to substrate at 37 °C, prior to the addition of A3A.

### RPA EMSA with linear or hairpin substrate

RPA was excluded from, or added to 25, 50, or 100 nM to 50 nM 5′-Cy5-labeled oligonucleotide substrate (hairpin-forming: oTM-814 or linear ssDNA: oTM-910), 20 mM Tris–HCl pH 7.5, 1 mM DTT, 1 mM EDTA, with 5 units of UDG. Binding reactions were incubated at room temperature for 15 min, before adding glycerol to 30%, and electrophoresing through a 6% native polyacrylamide gel on ice at 80 V for 1 h. Gels were imaged on a BioRad Chemidoc using the Cy5 setting. Percent substrate bound was determined by quantification of the intensity of size-shifted (bound) band relative to unbound substrate, using the "Volume" tool on BioRad’s Image Lab software version 6.0.1.

### In vitro transcription

The dsDNA substrate was prepared and purified as above using the oligos ALH287 and ALH288 (see Table S1). The gel band with the correct annealing product was determined by restriction digest as double-stranded sections contained AseI and PsiI sites that would only be cut if annealed properly (Fig. S3).

Deaminase activity assays with purified APOBEC3A (with concentrations as indicated), 50 units of T7 RNA Polymerase (NEB), 5 units of Uracil DNA glycosylase (NEB), 20 nM oligonucleotide substrate, 1 mM each of GTP, CTP, and UTP, 40 mM Tris HCl pH 8, 6 mM MgCl_2_, 1 mM DTT, and 1 mM EDTA, in a 20 μL volume were incubated for 24 h at 37 °C. Control reactions contained no T7 Polymerase or no A3A enzyme to ensure that deamination was transcription-dependent. In samples without enzyme, an equal volume of 50% glycerol was used instead. Reactions were terminated, products separated, and abundances quantified as described above for the measurement of deaminase activity. All full-length gel images of cytidine deaminase assays are presented in the supplementary information (Figs. S4–S8).

## Supplementary Information

Supplementary Information 1.

Supplementary Information 2.

## References

[1] Conticello SG (2008). The AID/APOBEC family of nucleic acid mutators. Genome Biol..

[2] Refsland EW, Harris RS (2013). The APOBEC3 family of retroelement restriction factors. Curr. Top. Microbiol. Immunol..

[3] Smith HC, Bennett RP, Kizilyer A, McDougall WM, Prohaska KM (2012). Functions and regulation of the APOBEC family of proteins. Semin. Cell. Dev. Biol..

[4] Burns MB, Leonard B, Harris RS (2015). APOBEC3B: Pathological consequences of an innate immune DNA mutator. Biomed. J..

[5] Chan K, Resnick MA, Gordenin DA (2013). The choice of nucleotide inserted opposite abasic sites formed within chromosomal DNA reveals the polymerase activities participating in translesion DNA synthesis. DNA Repair.

[6] Hoopes JI (2017). Avoidance of APOBEC3B-induced mutation by error-free lesion bypass. Nucleic Acids Res..

[7] Alexandrov LB (2013). Signatures of mutational processes in human cancer. Nature.

[8] Burns MB, Temiz NA, Harris RS (2013). Evidence for APOBEC3B mutagenesis in multiple human cancers. Nat. Genet..

[9] Nik-Zainal S (2012). Mutational processes molding the genomes of 21 breast cancers. Cell.

[10] Roberts SA (2013). An APOBEC cytidine deaminase mutagenesis pattern is widespread in human cancers. Nat. Genet..

[11] Roberts SA (2012). Clustered mutations in yeast and in human cancers can arise from damaged long single-strand DNA regions. Mol. Cell..

[12] Alexandrov LB (2020). The repertoire of mutational signatures in human cancer. Nature.

[13] Buisson R (2019). Passenger hotspot mutations in cancer driven by APOBEC3A and mesoscale genomic features. Science.

[14] Burns MB (2013). APOBEC3B is an enzymatic source of mutation in breast cancer. Nature.

[15] Starrett GJ (2016). The DNA cytosine deaminase APOBEC3H haplotype I likely contributes to breast and lung cancer mutagenesis. Nat. Commun..

[16] Chan K (2015). An APOBEC3A hypermutation signature is distinguishable from the signature of background mutagenesis by APOBEC3B in human cancers. Nat. Genet..

[17] Cortez LM (2019). APOBEC3A is a prominent cytidine deaminase in breast cancer. PLoS Genet..

[18] Periyasamy M (2015). APOBEC3B-mediated cytidine deamination is required for estrogen receptor action in breast cancer. Cell. Rep..

[19] Bogerd HP (2006). Cellular inhibitors of long interspersed element 1 and Alu retrotransposition. Proc. Natl. Acad. Sci. USA.

[20] Law EK (2020). APOBEC3A catalyzes mutation and drives carcinogenesis in vivo. J. Exp. Med..

[21] Petljak, M. *et al.* The APOBEC3A deaminase drives episodic mutagenesis in cancer cells. *BioRxiv*, 2021.02.14.431145 (2021).

[22] Ito F, Fu Y, Kao SA, Yang H, Chen XS (2017). Family-wide comparative analysis of cytidine and methylcytidine deamination by eleven human APOBEC proteins. J. Mol. Biol..

[23] Haradhvala NJ (2016). Mutational strand asymmetries in cancer genomes reveal mechanisms of DNA damage and repair. Cell.

[24] Morganella S (2016). The topography of mutational processes in breast cancer genomes. Nat. Commun..

[25] Nik-Zainal S (2016). Landscape of somatic mutations in 560 breast cancer whole-genome sequences. Nature.

[26] Seplyarskiy VB (2016). APOBEC-induced mutations in human cancers are strongly enriched on the lagging DNA strand during replication. Genome Res..

[27] Sakhtemani R (2019). Genome-wide mapping of regions preferentially targeted by the human DNA-cytosine deaminase APOBEC3A using uracil-DNA pulldown and sequencing. J. Biol. Chem..

[28] Logue EC (2014). A DNA sequence recognition loop on APOBEC3A controls substrate specificity. PLoS ONE.

[29] Pham P, Landolph A, Mendez C, Li N, Goodman MF (2013). A biochemical analysis linking APOBEC3A to disparate HIV-1 restriction and skin cancer. J. Biol. Chem..

[30] Silvas TV (2018). Substrate sequence selectivity of APOBEC3A implicates intra-DNA interactions. Sci. Rep..

[31] Love RP, Xu H, Chelico L (2012). Biochemical analysis of hypermutation by the deoxycytidine deaminase APOBEC3A. J. Biol. Chem..

[32] Thielen BK (2010). Innate immune signaling induces high levels of TC-specific deaminase activity in primary monocyte-derived cells through expression of APOBEC3A isoforms. J. Biol. Chem..

[33] Kohli RM (2010). Local sequence targeting in the AID/APOBEC family differentially impacts retroviral restriction and antibody diversification. J. Biol. Chem..

[34] Pham P, Bransteitter R, Petruska J, Goodman MF (2003). Processive AID-catalysed cytosine deamination on single-stranded DNA simulates somatic hypermutation. Nature.

[35] Bransteitter R, Pham P, Scharff MD, Goodman MF (2003). Activation-induced cytidine deaminase deaminates deoxycytidine on single-stranded DNA but requires the action of RNase. Proc. Natl. Acad. Sci. USA.

[36] Dickerson SK, Market E, Besmer E, Papavasiliou FN (2003). AID mediates hypermutation by deaminating single stranded DNA. J. Exp. Med..

[37] Chaudhuri J (2003). Transcription-targeted DNA deamination by the AID antibody diversification enzyme. Nature.

[38] Yu K, Roy D, Bayramyan M, Haworth IS, Lieber MR (2005). Fine-structure analysis of activation-induced deaminase accessibility to class switch region R-loops. Mol. Cell Biol..

[39] Qiao Q (2017). AID recognizes structured DNA for class switch recombination. Mol. Cell..

[40] Chaudhuri J, Khuong C, Alt FW (2004). Replication protein A interacts with AID to promote deamination of somatic hypermutation targets. Nature.

[41] Basu U (2011). The RNA exosome targets the AID cytidine deaminase to both strands of transcribed duplex DNA substrates. Cell.

[42] Diamond CP (2019). AID, APOBEC3A and APOBEC3B efficiently deaminate deoxycytidines neighboring DNA damage induced by oxidation or alkylation. Biochim. Biophys. Acta Gen. Subj..

[43] Carpenter MA (2012). Methylcytosine and normal cytosine deamination by the foreign DNA restriction enzyme APOBEC3A. J. Biol. Chem..

[44] Wijesinghe P, Bhagwat AS (2012). Efficient deamination of 5-methylcytosines in DNA by human APOBEC3A, but not by AID or APOBEC3G. Nucleic Acids Res..

[45] Adolph MB, Love RP, Feng Y, Chelico L (2017). Enzyme cycling contributes to efficient induction of genome mutagenesis by the cytidine deaminase APOBEC3B. Nucleic Acids Res..

[46] Jalili P (2020). Quantification of ongoing APOBEC3A activity in tumor cells by monitoring RNA editing at hotspots. Nat. Commun..

[47] Sharma S, Baysal BE (2017). Stem-loop structure preference for site-specific RNA editing by APOBEC3A and APOBEC3G. PeerJ.

[48] Sharma S (2015). APOBEC3A cytidine deaminase induces RNA editing in monocytes and macrophages. Nat. Commun..

[49] Kouno T (2017). Crystal structure of APOBEC3A bound to single-stranded DNA reveals structural basis for cytidine deamination and specificity. Nat. Commun..

[50] Shi K (2017). Structural basis for targeted DNA cytosine deamination and mutagenesis by APOBEC3A and APOBEC3B. Nat. Struct. Mol. Biol..

[51] McDaniel YZ (2020). Deamination hotspots among APOBEC3 family members are defined by both target site sequence context and ssDNA secondary structure. Nucleic Acids Res..

[52] Green AM (2016). APOBEC3A damages the cellular genome during DNA replication. Cell Cycle.

[53] Hoopes JI (2016). APOBEC3A and APOBEC3B preferentially deaminate the lagging strand template during DNA replication. Cell. Rep..

[54] Saini N (2017). APOBEC3B cytidine deaminase targets the non-transcribed strand of tRNA genes in yeast. DNA Repair.

[55] Sui Y (2020). Analysis of APOBEC-induced mutations in yeast strains with low levels of replicative DNA polymerases. Proc. Natl. Acad. Sci. USA.

[56] Lada AG (2015). Disruption of transcriptional coactivator sub1 leads to genome-wide re-distribution of clustered mutations induced by APOBEC in active yeast genes. PLoS Genet..

[57] Taylor BJ, Wu YL, Rada C (2014). Active RNAP pre-initiation sites are highly mutated by cytidine deaminases in yeast, with AID targeting small RNA genes. Elife.

[58] Ramiro AR, Stavropoulos P, Jankovic M, Nussenzweig MC (2003). Transcription enhances AID-mediated cytidine deamination by exposing single-stranded DNA on the nontemplate strand. Nat. Immunol..

[59] Mitra M (2014). Structural determinants of human APOBEC3A enzymatic and nucleic acid binding properties. Nucleic Acids Res..

[60] Stewart JA, Schauer G, Bhagwat AS (2020). Visualization of uracils created by APOBEC3A using UdgX shows colocalization with RPA at stalled replication forks. Nucleic Acids Res..

[61] Blackwell LJ, Borowiec JA (1994). Human replication protein A binds single-stranded DNA in two distinct complexes. Mol. Cell Biol..

[62] Kim C, Snyder RO, Wold MS (1992). Binding properties of replication protein A from human and yeast cells. Mol. Cell Biol..

[63] Zuo Y, Steitz TA (2015). Crystal structures of the *E. coli* transcription initiation complexes with a complete bubble. Mol. Cell..

[64] Kazanov MD (2015). APOBEC-induced cancer mutations are uniquely enriched in early-replicating, gene-dense, and active chromatin regions. Cell Rep..

[65] Bhagwat AS (2016). Strand-biased cytosine deamination at the replication fork causes cytosine to thymine mutations in *Escherichia coli*. Proc. Natl. Acad. Sci. USA.

[66] Wong L, Vizeacoumar FS, Vizeacoumar FJ, Chelico L (2021). APOBEC1 cytosine deaminase activity on single-stranded DNA is suppressed by replication protein A. Nucleic Acids Res..

[67] Lada AG (2011). Replication protein A (RPA) hampers the processive action of APOBEC3G cytosine deaminase on single-stranded DNA. PLoS ONE.

[68] Toledo LI (2013). ATR prohibits replication catastrophe by preventing global exhaustion of RPA. Cell.

[69] Cramer P (2008). Structure of eukaryotic RNA polymerases. Annu. Rev. Biophys..

[70] Henricksen LA, Umbricht CB, Wold MS (1994). Recombinant replication protein A: Expression, complex formation, and functional characterization. J. Biol. Chem..

