# Supplementary material for: Single-stranded DNA binding proteins influence APOBEC3A substrate preference
Source: Sci Rep. 2021 Oct 25;11:21008. doi: 10.1038/s41598-021-00435-y (PMC8546098; doi:10.1038/s41598-021-00435-y)
Supplement: Supplementary file 1 — Supplementary Information 1. [file 41598_2021_435_MOESM1_ESM.pdf]

Figure S1

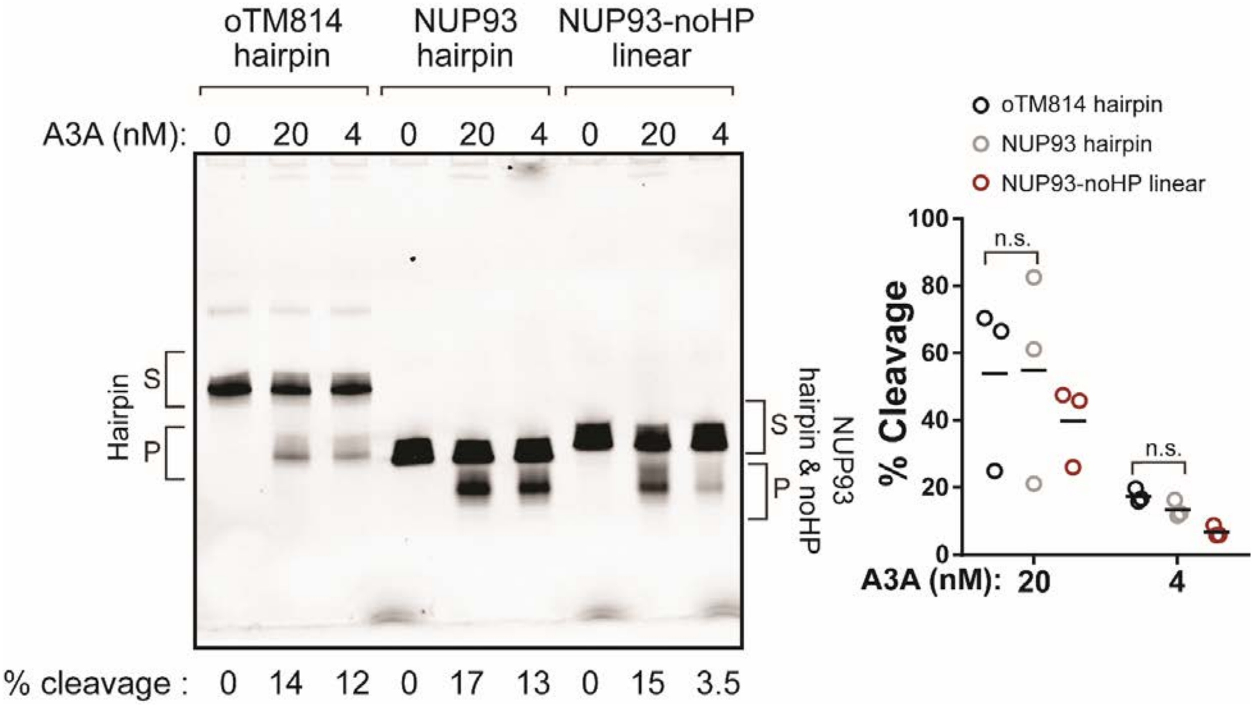

Fig. S1: Sequence composition of hairpin substrates does not significantly affect A3A activity. Deaminase assay of 20 nM, 4 nM, or no A3A with 250 nM substrates incubated for 30 min at 37˚C before processing as in Fig. 1C. S denotes substrate band; P denotes the product band. The percent cleavage for three replicate experiments was quantified and compared by ratio paired t-test. Horizontal bar indicates the mean value. n.s. indicates differences were “not significant.”

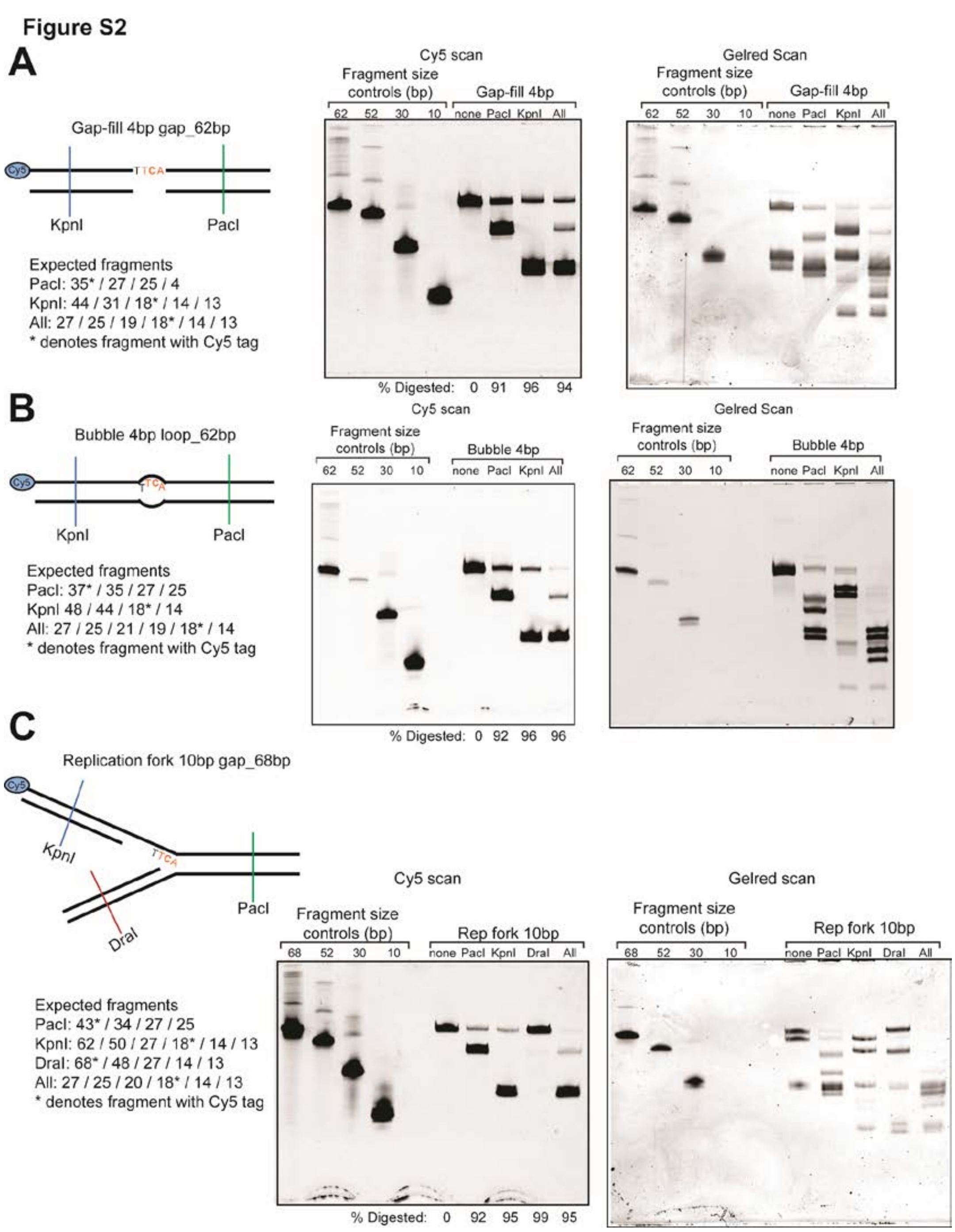

Fig. S2: Confirmation of annealed substrates. Representative PacI, KpnI, and DraI restriction digests of annealed substrates: A) ssDNA gap, B) bubble, C) replication fork substrates. Pictured are Cy5 and GelRed scans of 15% denaturing polyacrylamide gels with separated digestion products.

Figure S3

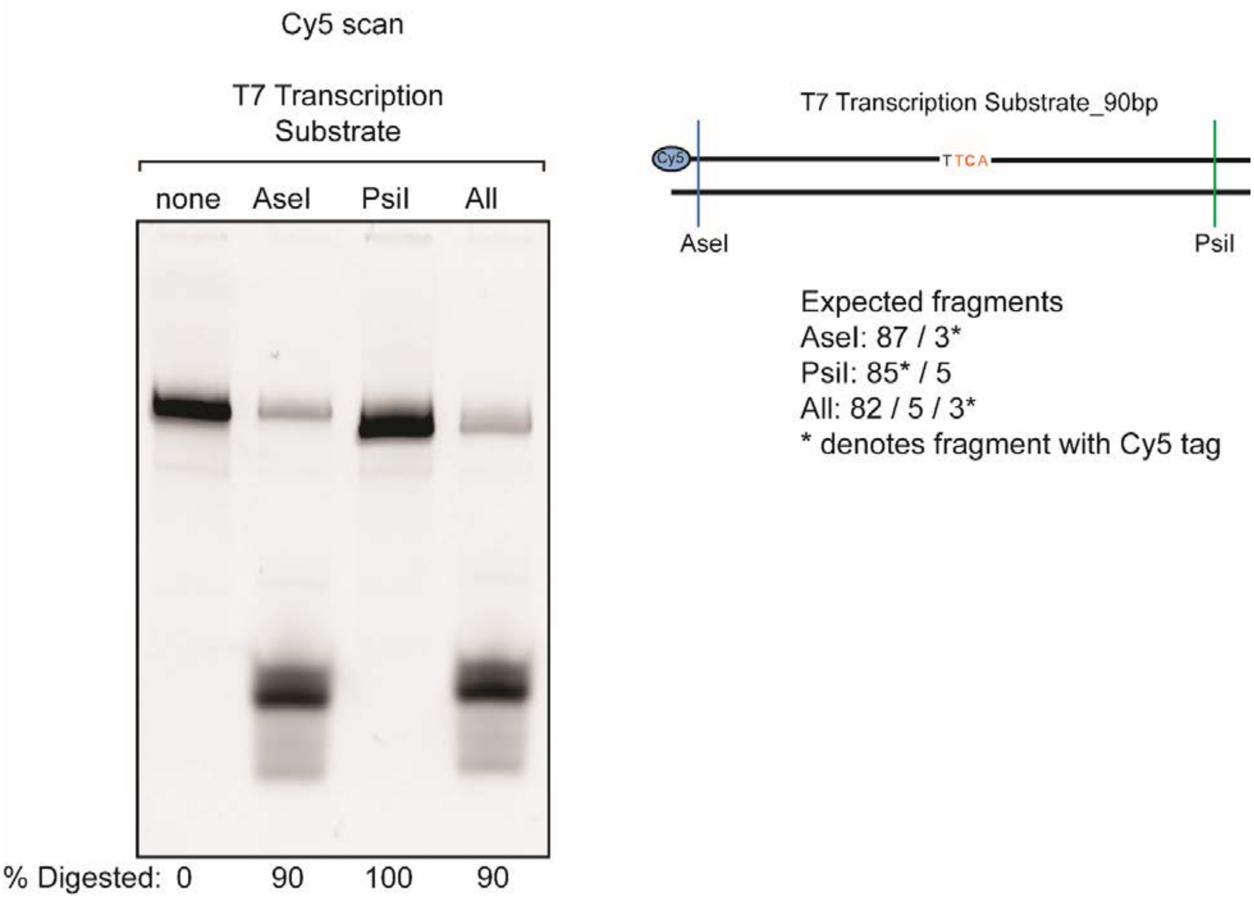

Fig. S3: Confirmation of annealed dsDNA substrate for *in vitro* transcription assay. Representative AseI and PsiI restriction digests of annealed substrate. Pictured is the Cy5 scan of a 15% denaturing polyacrylamide gel with separated digestion products.

Figure S4

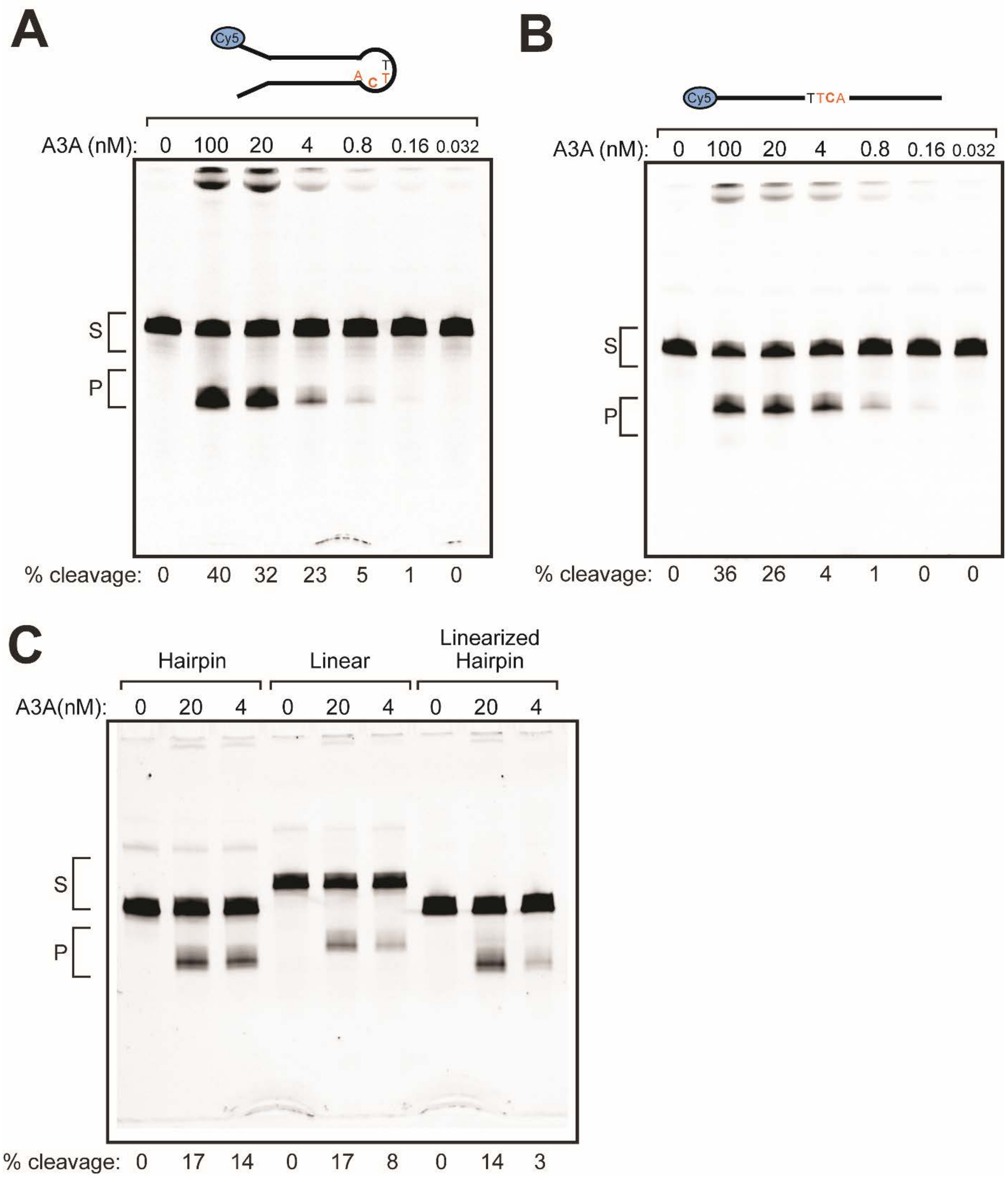

Fig. S4: Full length gel images for Fig. 1. Gel images without vertical cropping as in (A) Fig. 1C upper panel, (B) Fig. 1C lower panel, and (C) Fig. 1D.

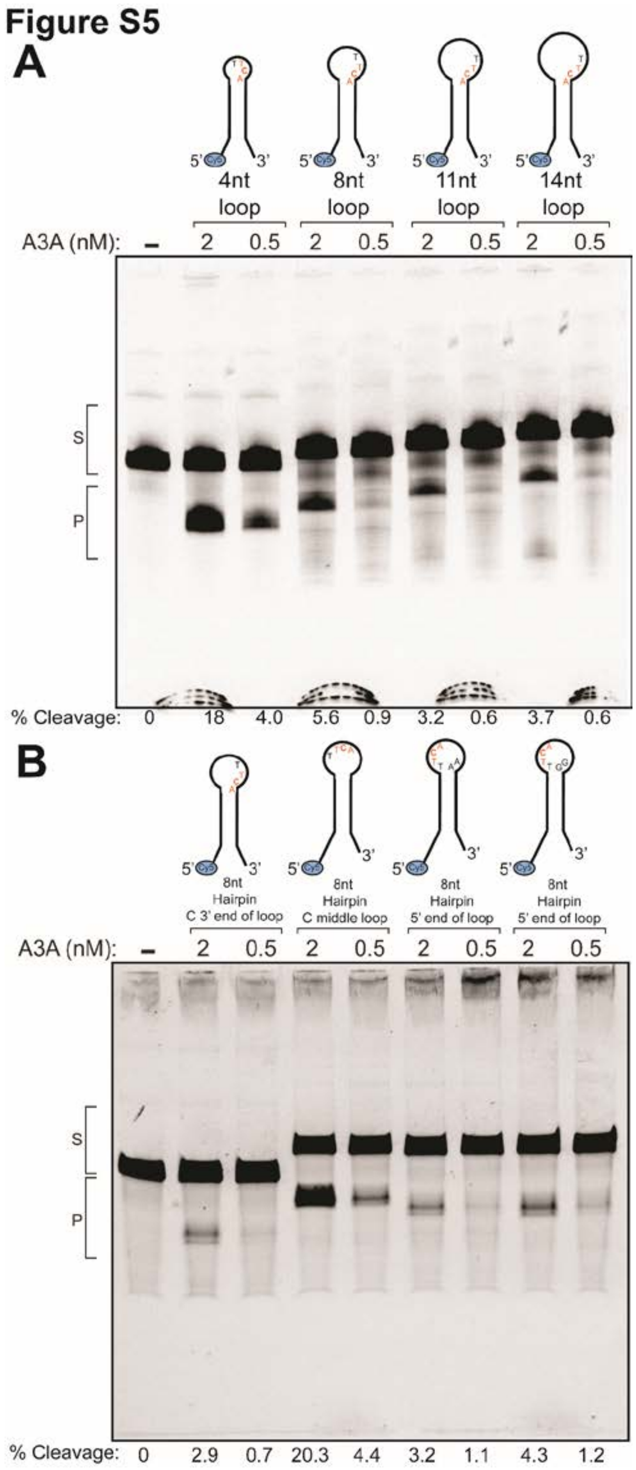

Fig. S5: Full length gel images for Fig. 2. Gel images without vertical cropping as in (A) Fig. 2A and (B) Fig. 2B.

Figure S6

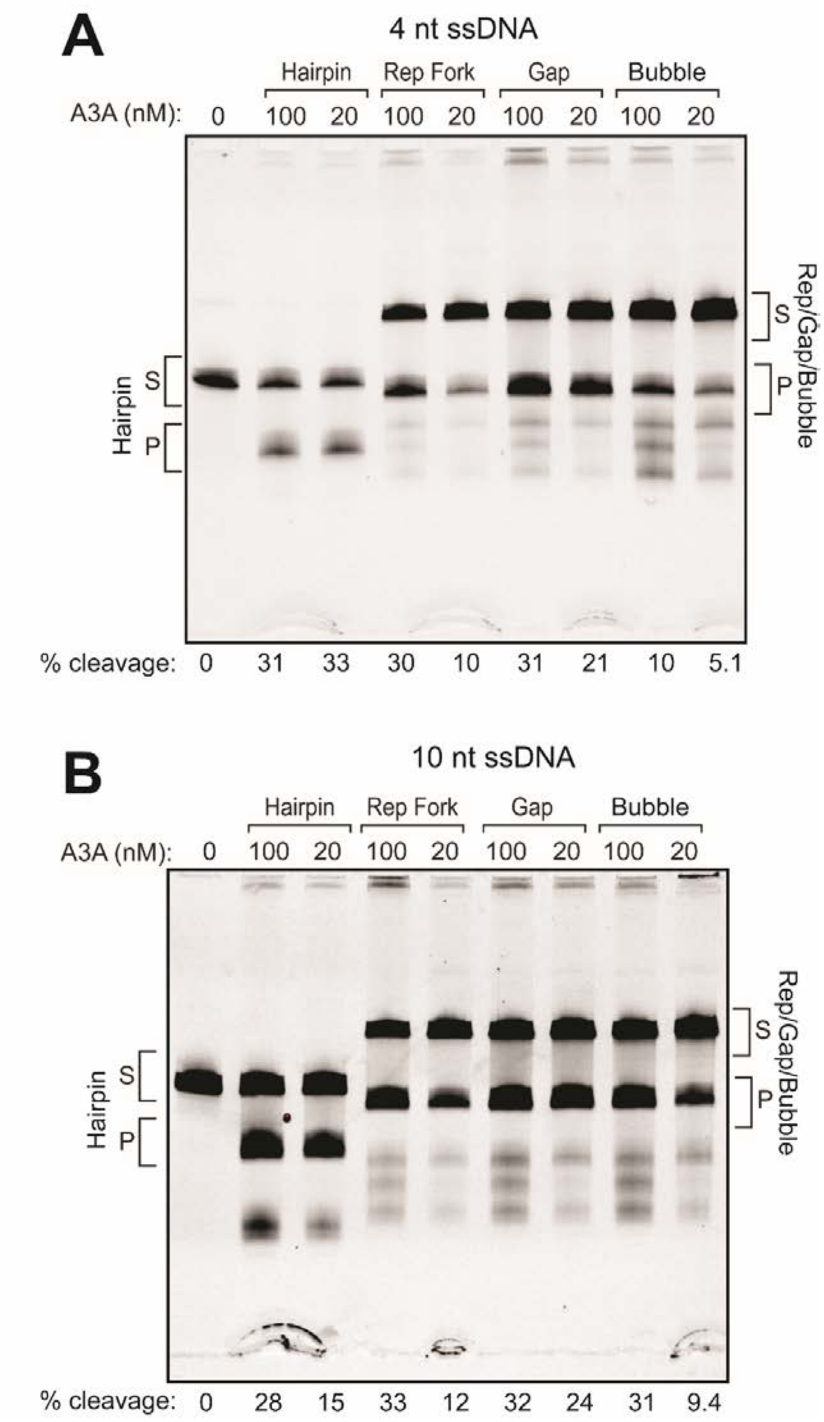

Fig. S6: Full length gel images for Fig. 3. Gel images without vertical cropping as in (A) Fig. 3B and (B) Fig. 3D.

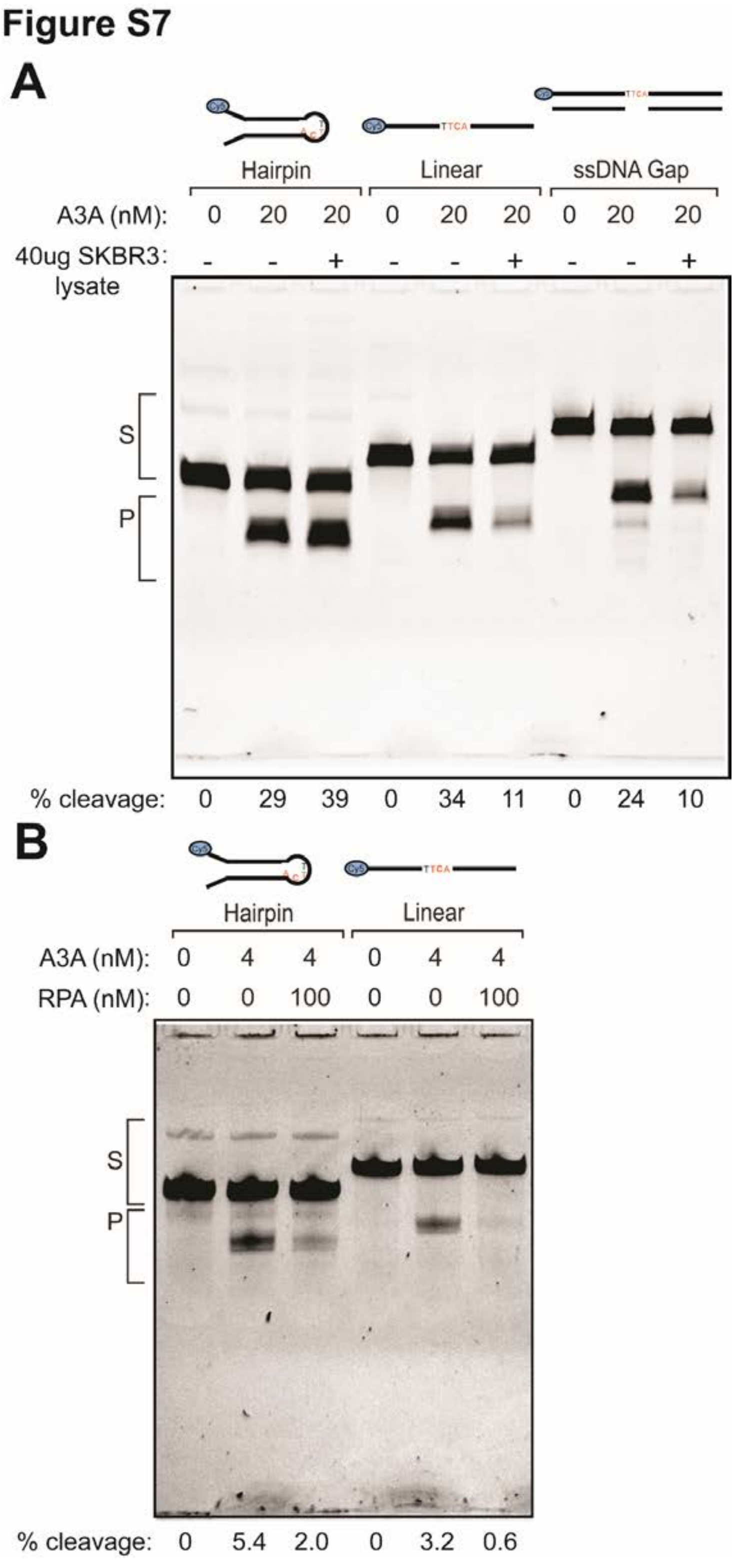

Fig. S7: Full length gel images for Fig. 4. Gel images without vertical cropping as in (A) Fig. 4A and (B) Fig. 4D.

Figure S8

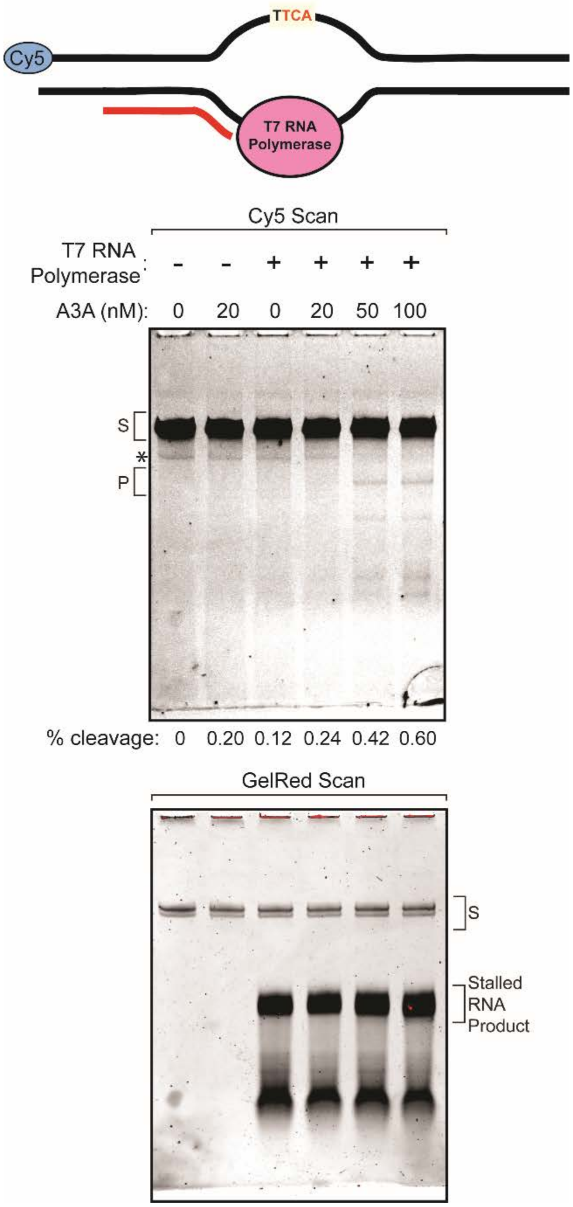

Fig. S8: Full length gel images for Fig. 5. Gel images without vertical cropping as in Fig. 5.

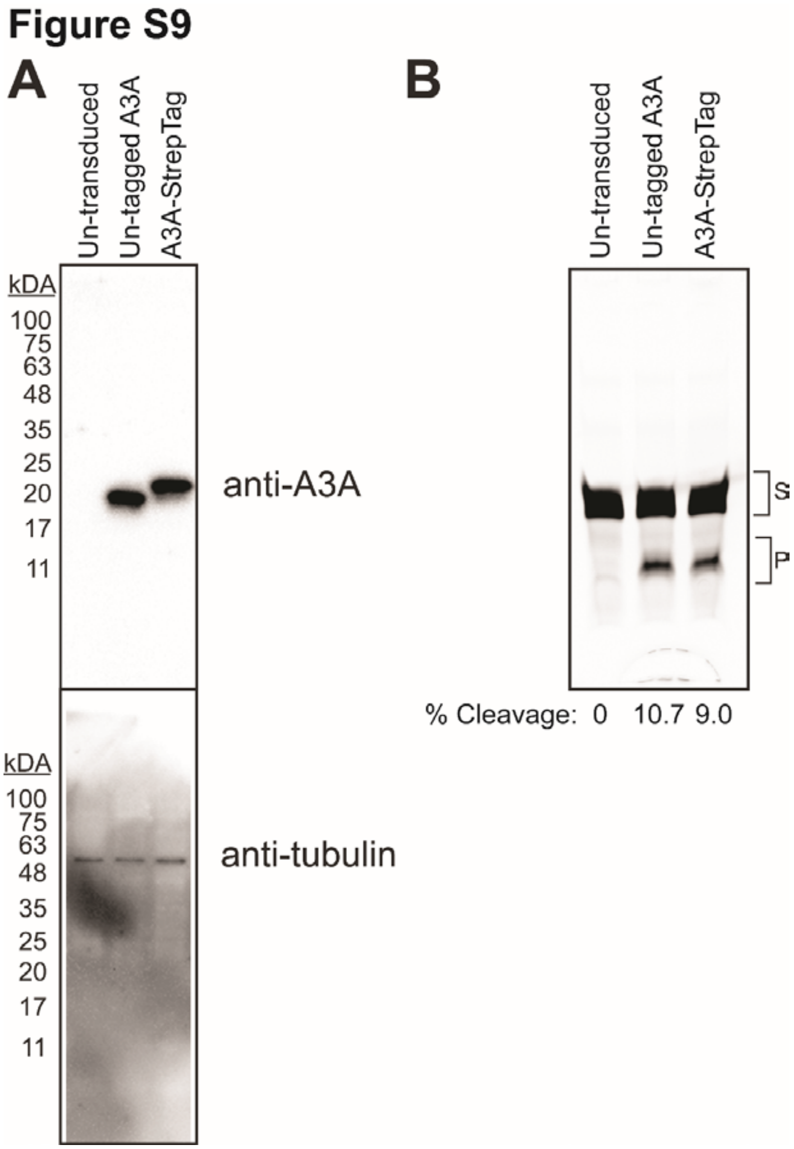

Fig. S9: Deaminase activity of un-tagged and C-terminally Strep-tagged A3A. (A) Western analysis of the induced expression of un-tagged and C-terminally Strep-tagged A3A in extracts from HEK293T cells. Blots were probed with anti-A3A and anti-tubulin antibodies. (B) 20 µg of whole cell extracts shown in (A) were incubated with 1 µM hairpin substrate containing a 4 bp ssDNA loop for 5 min at 37ºC. S indicates substrate. P indicates product. Percent cleavage of the substrate (indicative of A3A deaminase activity) was quantified. Shown is a representative image of two replicate experiments.

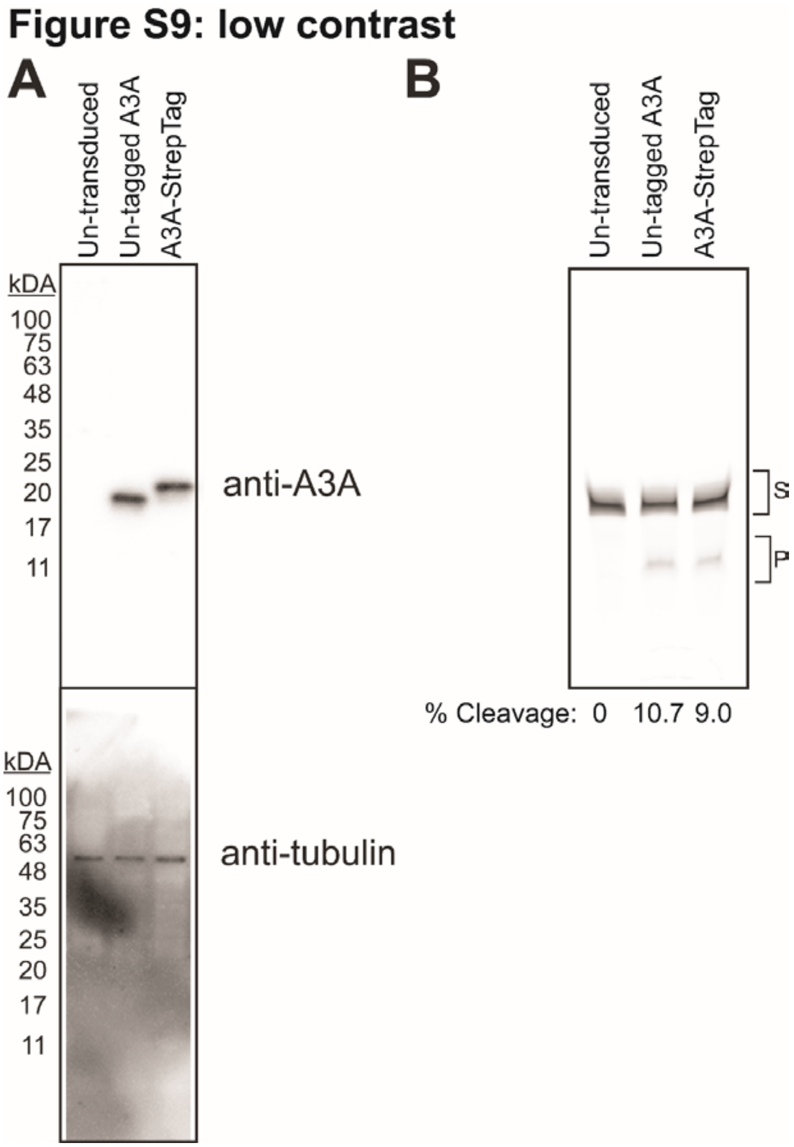

Fig. S9: Deaminase activity of un-tagged and C-terminally Strep-tagged A3A. (A) Western analysis of the induced expression of un-tagged and C-terminally Strep-tagged A3A in extracts from HEK293T cells. Blots were probed with anti-A3A and anti-tubulin antibodies. (B) 20 µg of whole cell extracts shown in (A) were incubated with 1 µM hairpin substrate containing a 4 bp ssDNA loop for 5 min at 37ºC. S indicates substrate. P indicates product. Percent cleavage of the substrate (indicative of A3A deaminase activity) was quantified. Shown is a representative image of two replicate experiments.

Table S1. Oligos used in this study
